# The effect of ivabradine therapy on dilated cardiomyopathy patients with congestive heart failure: a systematic review and meta-analysis

**DOI:** 10.3389/fcvm.2023.1149351

**Published:** 2023-10-17

**Authors:** Juntao Yang, Tingting Lv, Jiedong Zhou, Hui Lin, Bingjie Zhao, Haifei Lou, Hanxuan Liu, Tao Zhang, Hangyuan Guo, Jufang Chi

**Affiliations:** ^1^ School of Medicine, Shaoxing University, Shaoxing, China; ^2^ Ningbo Medical Center Lihuili Hospital (Lihuili Hospital Affiliated to Ningbo University), Ningbo, China; ^3^ Department of Cardiology, Shaoxing People’s Hospital, Shaoxing, China; ^4^ Department of Cardiology, Zhuji People’s Hospital, Zhuji, China

**Keywords:** ivabradine, dilated cardiomyopathy, heart failure, heart rate, cardiac function

## Abstract

**Background:**

Ivabradine improves cardiac function in patients with heart failure, but its effect on dilated cardiomyopathy (DCM) remains unclear. We performed a systematic review and meta-analysis to study the efficacy and potential mechanisms of ivabradine's effect on cardiac function and prognosis in patients with DCM.

**Methods:**

We searched PubMed, Cochrane Library, Embase, Web of Science, and four registers through September 28, 2022. All controlled trials of ivabradine for the treatment of DCM with congestive heart failure were included. Articles were limited to English, with the full text and necessary data available. We performed random- or fixed effects meta-analyses for all included outcome measures and compared the effect sizes for outcomes in patients treated with and without ivabradine. The quality of the studies was assessed using the Cochrane risk-of-bias tool for randomized trials (RoB2.0).

**Findings:**

Five trials with 357 participants were included. The pooled risk ratio was 0.48 [95% confidence interval (CI) (0.18, 1.25)] for all-cause mortality and 0.38 [95% CI (0.12, 1.23)] for cardiac mortality. The pooled mean difference was −15.95 [95% CI (−19.97, −11.92)] for resting heart rate, 3.96 [95% CI (0.99, 6.93)] for systolic blood pressure, 2.93 [95% CI (2.09, 3.77)] for left ventricular ejection fraction, −5.90 [95% CI (−9.36, −2.44)] for left ventricular end-systolic diameter, −3.41 [95% CI (−5.24, −1.58)] for left ventricular end-diastolic diameter, −0.81 [95% CI (−1.00, −0.62)] for left ventricular end-systolic volume, −0.67 [95% CI (−0.86, −0.48)] for left ventricular end-diastolic volume, −11.01 [95% CI (−19.66, −2.35)] for Minnesota Living with Heart Failure score, and −0.52 [95% CI (−0.73, −0.31)] for New York Heart Association class.

**Interpretation:**

Ivabradine reduces heart rate and ventricular volume, and improves cardiac function in patients with DCM, but showed no significant effect on the prognosis of patients.

## Introduction

Dilated cardiomyopathy (DCM) is a non-ischemic myocardial disease with structural and functional abnormalities characterized by left or bilateral ventricular dilation and systolic dysfunction in the absence of coronary heart disease (CHD), hypertension, valvular disease, and congenital heart disease (1). DCM should not be regarded as a single disease entity but as a non-specific phenotype that is the final common response of the myocardium to a variety of pathogenic factors (2, 3). Its clinical features are mainly congestive heart failure (CHF) and various arrhythmias (1). In a 2013 review based on recent clinical trials and associated data, the estimated prevalence of DCM was >1 per 250 people (4). Three-year treated mortality rates remain high at 12%–20%, with death usually due to heart failure (HF) or sudden cardiac death caused by ventricular arrhythmia (5).

At present, DCM treatment mainly includes angiotensin-converting enzyme inhibitors, angiotensin receptor antagonists, β-blockers, aldosterone receptor antagonists, devices, and mechanical circulatory support (6). Although the 5-year survival rate has significantly improved with improvements in treatment methods, there is still a big gap compared with other cardiovascular diseases. Ivabradine is a selective *I*_f_ current inhibitor that lowers heart rate by reducing the rate of phase 4 spontaneous depolarization of autorhythmic cells (7). The relationship between heart rate and the prognosis of HF has long been demonstrated in clinical studies (8). Because ivabradine has no direct effect on myocardial contractility and conductivity (7), and rarely blocks other receptors or channels outside the heart to cause other adverse reactions, it is far more applicable and safe than β-blockers, which also reduce heart rate. Due to its anti-myocardial ischemic and cardiac function-improving effects, it is often used to treat CHD and systolic HF in clinical practice (9).

The effectiveness of ivabradine in DCM has been increasingly reported in the last 10 years (10), but it is only currently approved by the US Food and Drug Administration and the European Medicines Agency for the treatment of stable angina pectoris and HF with reduced ejection fraction (11, 12). Its potential role in treating DCM has not been clearly recognized. Therefore, we performed a systematic review and meta-analysis to evaluate the efficacy of ivabradine in reducing heart rate and improving cardiac function and prognosis in patients with DCM. Then we will further explore its specific mechanisms and efficacy in the treatment of DCM compared to general HF.

## Methods

The reporting of this systematic review was guided by the standards of the Preferred Reporting Items for Systematic Review and Meta-Analysis (PRISMA) 2020 statement (13, 14).

### Literature search strategy

We searched four databases (Embase, PubMed, Web of Science, and Cochrane Library) for published literature and four clinical trial registries [ClinicalTrials.gov (www.clinicaltrials.gov), Chinese Clinical Trials Registry (www.chictr.org.cn), EU Clinical Trials Register (www.clinicaltrialsregister.eu) and University Hospital Medical Information Network Clinical Trials Registry (www.umin.ac.jp/ctr)] for ongoing trials until September 28, 2022, to identify potential studies of ivabradine in treating DCM. Medical Subject Headings and Emtree were used for search terms. The following terms were included in the title or abstract: (“cardiomyopathy, dilated” or “dilated cardiomyopathies” or “dilated cardiomyopathy” or “familial idiopathic cardiomyopathy” or “congestive cardiomyopathy” or “congestive cardiomyopathies”) and (“ivabradine” or “corlanor”). Reviews and conference abstracts were also searched. The search was limited to English language and human studies. If there were articles and studies for which complete information was not available, we asked the authors for unpublished content via email. When the systematic review was completed, we ran a search again to ensure that no new studies were missed. The literature search strategy was formulated according to the PRISMA-S (Preferred Reporting Items for Systematic reviews and Meta-Analyses literature search extension) (15).

Three authors (J.Y., T.L. and J.Z.) independently reviewed the titles and abstracts of all records and screened the full-text articles for inclusion. Duplicates were identified automatically using EndNote's duplicate identification strategy and then manually removed. Any inconsistencies were resolved through discussions among the three authors.

### Eligibility criteria

The inclusion criteria were as follows: (1) patients diagnosed with DCM with CHF; (2) the experimental group was treated with ivabradine alone or in combination with other treatments compared with a control group; (3) report at least one of the following outcome measures: resting heart rate (RHR), left ventricular ejection fraction (LVEF), left ventricular end-systolic diameter (LVESD), left ventricular end-diastolic diameter (LVEDD), left ventricular end-systolic volume (LVESV), left ventricular end-diastolic volume (LVEDV), systolic blood pressure (SBP), Minnesota Living with Heart Failure (MLWHF) score, New York Heart Association (NYHA) class, all-cause mortality, and cardiac mortality; and (4) the study type was two-arm interventional trial. The exclusion criteria were as follows: (1) studies not written in English; (2) studies without sufficient data for meta-analysis; (3) studies with duplicate data or repeat analysis; (4) studies for which the full text could not be obtained; and (5) animal studies, review articles and case reports.

### Data extraction

The information and data of the included studies were independently extracted by two authors (J.Y. and J.Z.) and checked by a third author (T.L.). The extracted data included study characteristics (first author's last name, year of publication, country, sample size, follow-up duration, dose of ivabradine, and endpoints) and patient characteristics (sex, age, NYHA class, LVEF, RHR, comorbidities and specific medications). The data extracted also included means for each outcome measure (shown in the inclusion criteria), standard deviations of means, and sample size for both groups. In addition, all-cause and cardiac mortality rates were collected for both groups. In the case of missing data, unpublished data were sought from the author of the article via email.

### Risk-of-bias and quality assessment

The risk-of-bias assessment for the five studies included in this analysis was done independently by two authors (J.Y. and J.Z.) using the recently revised Cochrane risk-of-bias tool for randomized trials (RoB2.0) (16). This tool is structured into five bias domains: bias arising from the randomization process, bias due to deviations from intended interventions, bias due to missing outcome data, bias in measurement of the outcome, and bias in selection of the reported result. For each domain, a series of signaling questions with answers (yes, probably yes, no information, probably no, no) determine the risk of bias (low risk, some concerns, and high risk). We used the excel tool provided by the MRC Network of Hubs for Trials Methodology Research to perform the specific assessment process (https://sites.google.com/site/riskofbiastool/welcome/rob-2-0-tool/current-version-of-rob-2). Any disagreements were resolved through discussion with a third author (T.L.).

### Statistical analysis

The intervention of the experimental group in each study was ivabradine combined with general treatment; therefore, every study was eligible for each planned synthesis. Tables and forest plots were used to display the results of the statistical analysis. Dichotomous variables were analyzed using risk ratios (RR) with 95% confidence intervals (CIs), whereas continuous variables were analyzed using weighted mean differences (WMD) or standardized mean differences (SMD). Because LVESV and LVEDV were not measured in exactly the same way in each study, SMD was used as a summary statistic, and WMD was used for the remaining continuous variables. Cochran's *Q*-test and *I*^2^ statistic were used to assess the heterogeneity of the included studies, and the heterogeneity tests mainly referred to *I*^2^ values due to the small number of included studies. *I*^2^ values >50% indicated high heterogeneity. Pooled analyses were performed using fixed-effect models, whereas random-effect models were used when there was a high degree of heterogeneity between studies. Because the incidence of outcome events was particularly low for dichotomous variables, and heterogeneity across studies was not large, we chose the Mantel–Haenszel analysis method. The inverse variance method was used for the statistical analysis of all continuous variables. Because there was little difference in baseline data for NYHA class between studies and only post-follow-up data were provided, we used post-follow-up data for analysis. Change-from-baseline data were used for the remaining continuous variables. The missing standard deviations of change-from-baseline data were calculated using the correlation coefficients.

Subgroup analyses were performed to examine whether baseline LVEF, follow-up duration, and age affected the effect size and heterogeneity. Within-study contrasts were also performed because data on subsets of participants were available in some studies. Sensitivity analyses (excluding 1 study at a time) were performed to determine the stability of the overall treatment effect. Cumulative analyses were used to assess the effect of publication time on the pooled estimates. In addition, Egger's linear regression test was used to assess publication bias, and the stability of the pooled results of outcome measures with significant publication bias (*p* < 0.05) was assessed using the trim-and-fill method. Statistical analyses were performed using the Review Manager software (version 5.4.1, The Cochrane Collaboration, 2020) and Stata software (version 12.0; Stata Corp., College Station, Texas, USA). All *p*-values were 2-tailed, and *p*-values < 0.05 were considered to be statistically significant.

### Quality of the evidence

We employed the methods and recommendations described in the “Cochrane Handbook for Systematic Reviews of Interventions” to carry out this item (17). Three authors (J.Y., T.L. and J.Z.) independently assessed the quality of evidence for all the outcome measures. We used the five GRADE considerations (study limitations, inconsistency, indirectness, imprecision, and publication bias) to assess the quality of the evidence related to the studies that contributed data to the meta-analyses of the pre-specified outcomes (Supplementary Table S1). We considered the following criteria to upgrade the quality of the evidence as appropriate: large effect, dose-response gradient, and plausible confounding effect. The assessment of the evidence quality was categorized as high, moderate, low, or very low. To compile the “Summary of Findings” tables, we employed the GRADEpro software (GRADEpro GDT 2015). All decisions to downgrade or upgrade the quality of the studies have been thoroughly justified through footnotes.

## Results

### Characteristics of included studies

**Figure 1:**
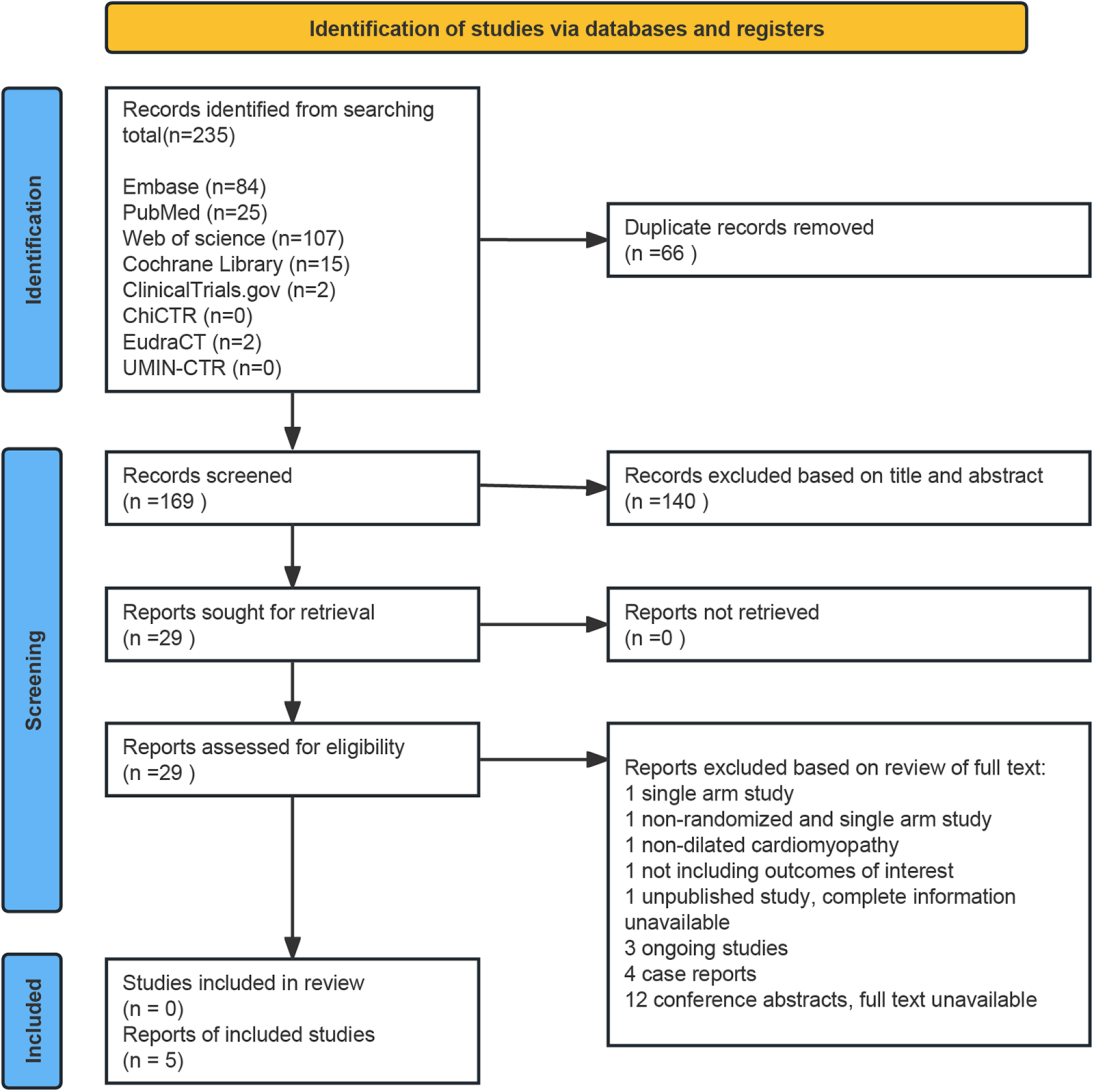
Flow diagram of the studies selection process in the systematic review.

The process of the literature search is outlined in Figure 1. Our initial search identified 235 records, leaving 169 records after removing duplicates. A total of 140 articles were excluded after the title and abstract screening. Five studies were finally included by reviewing the full texts of the remaining 29 articles, and we briefly listed the reasons for exclusion in the flow diagram. The screening process strictly followed pre-established inclusion and exclusion criteria.

The characteristics of the included studies are summarized in Table 1. Five studies (10, 18–21) involved a total of 357 participants. One study was conducted in 47 centers in 16 countries, and the participants were all minors. The follow-up duration refers to the maximum time from the start of treatment to the outcome measurement. For the main outcome measures, the follow-up duration of the five studies extended up to 12 months. In all five studies, ivabradine was titrated using a starting low dose method and finally maintained at either the target dose or the maximum tolerated dose. We recorded the final average dose for the experimental groups. All studies reported RHR and LVEF, while the remaining outcome measures were only reported in some of the studies.

**Table 1:** Characteristics of included studies.

First author, year	Mansour et al. (18)	Abdel-Salam et al. (19)	Bonnet et al. (10)	Chandh Raja et al. (20)	Adorisio et al. (21)
Country	Egypt	Egypt	16 countries in Europe and America	India	Italy
Sample size	Iva:30, Ctrl:23	Iva:20, Ctrl:23	Iva:74, Ctrl:42	Iva:63, Ctrl:62	Iva:9, Ctrl:11
Male/Female	Iva:18/12, Ctrl:14/9	Iva:10/10, Ctrl:13/10	Iva:39/35, Ctrl:25/17	Iva:35/28, Ctrl:37/25	Iva:9/0, Ctrl:11/0
Age (mean; years)	Iva:47, Ctrl:52	Iva:49.1, Ctrl:52.3	Iva:5.8, Ctrl:5.8	Iva:48.9, Ctrl:45.4	Iva:22, Ctrl:19
NYHA class	Iva:7Ⅱ/22Ⅲ/1Ⅳ, Ctrl:3Ⅱ/14Ⅲ/6Ⅳ	Iva:6Ⅱ/12Ⅲ/2Ⅳ, Ctrl:5Ⅱ/14Ⅲ/4Ⅳ	Iva:59Ⅱ/12Ⅲ/3Ⅳ, Ctrl:34Ⅱ/6Ⅲ/2Ⅳ	Iva:3.3 ± 0.5, Ctrl:3.2 ± 0.4	Not reported
RHR (mean; bpm)	Iva:96, Ctrl:84	Iva:85, Ctrl:84	Iva:102, Ctrl:100	Iva:95, Ctrl:95	Iva:90, Ctrl:100
LVEF (mean; %)	Iva:30.2, Ctrl:32.3	Iva:32, Ctrl:32	Iva:32, Ctrl:35	Iva:26, Ctrl:26.7	Iva:24, Ctrl:30
Does of ivabradine	11.6 ± 3.4 mg/day	13.6 mg/day	children ages 6–12 months: 0.29 ± 0.17 mg/kg/day	13.3 ± 2.3 mg/day	15 mg/day
			children ages 1–3 years: 0.44 ± 0.2 mg/kg/day		
			children ages 3–18 years, <40 kg: 0.3 ± 0.16 mg/kg/day		
			children ages 3–18 years, >40 kg: 11.82 ± 9.3 mg/day		
Follow-up (months)	3 and 13.5	3	End of Titration, 6 and 12	3 and 6	12
Endpoints	RHR, LVEF, MLWHF score, LVEDV, LVESV, LVEDD, LVESD, All-cause mortality, Cardiac mortality	RHR, LVEF, MLWHF score, LVEDV, LVESV, LVEDD, LVESD, SBP, NYHA class, All-cause mortality, Cardiac mortality	RHR, LVEF, LVEDV, LVESV, All-cause mortality, Cardiac mortality	RHR, LVEF, MLWHF score, LVEDV, LVESV, LVEDD, LVESD, SBP, NYHA class, All-cause mortality, Cardiac mortality	RHR, LVEF, LVEDD, SBP
Hypertension (%)	Iva:17, Ctrl:17	Iva:25, Ctrl:17.4	Not reported	Iva:32.3, Ctrl:32.8	Not reported
Diabetes (%)	Iva:26, Ctrl:22	Iva:20, Ctrl:21.7	Not reported	Iva:32.3, Ctrl:36.1	Not reported
Beta-blocker use	Iva:30, Ctrl:23	Iva:20, Ctrl:23	Iva:59, Ctrl:29	Iva:63, Ctrl:62	Iva:9, Ctrl:11
ACEI use	Not reported	Iva:20, Ctrl:23	Iva:70, Ctrl:39	Iva:63, Ctrl:62	Iva:9, Ctrl:11
Aldosterone antagonist use	Not reported	Iva:20, Ctrl:23	Iva:63, Ctrl:28	27% of patients	Iva:7, Ctrl:6
Digitalis use	Not reported	Not reported	Iva:39, Iva:19	67% of patients	Iva:2, Ctrl:3

Iva, ivabradine; Ctrl, control; RHR, resting heart rate; LVEF, left ventricular ejection fraction; MLWHF score, Minnesota Living with Heart Failure score; LVEDV, left ventricular end-diastolic volume; LVESV, left ventricular end-systolic volume; LVEDD, left ventricular end-diastolic diameter; LVESD, left ventricular end-systolic diameter; SBP, systolic blood pressure; NYHA class, New York Heart Association class; ACEI, angiotensin converting enzyme inhibitors.

### Quality assessment of included studies

**Figure 2:**
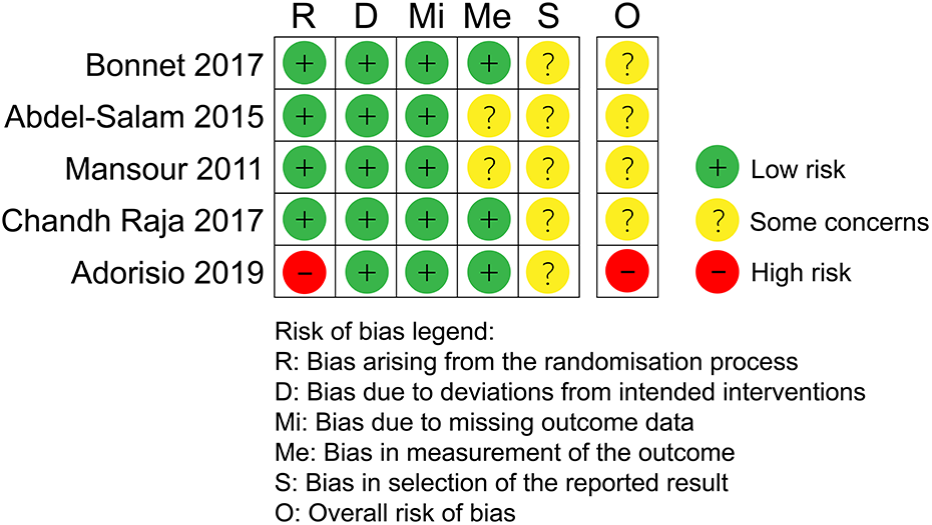
Risk of bias assessment using the Risk of Bias 2.0 tool.

The risk of bias in the included studies is shown in Figure 2. All studies had a low risk of bias with respect to the deviation from the intended intervention and missing outcome data. Except for Adorisio et al.'s study (21), which had a high risk of bias in the randomization process, all other studies had a low risk in this regard. Because none of the included studies provided a study plan, we were unable to assess for selective reporting of results; therefore, there are some concerns with respect to the selection of the reported result. Two studies (18, 19) had some concerns with respect to the measurement of outcomes because it was unclear whether some studies used the same measuring method for different outcome measures. Finally, according to the evaluation criteria, four studies (10, 18–20) had some concerns regarding the overall risk of bias, while one study (21) had a high overall risk of bias.

### Main efficacy of meta-analysis

The number of patients involved in each outcome measure is presented in the “Summary of Findings” tables (Supplementary Table S2), while the risk of bias is detailed in the “Quality Assessment of Included Studies” and will not be repeated here.

#### Resting heart rate

**Figure 3:**
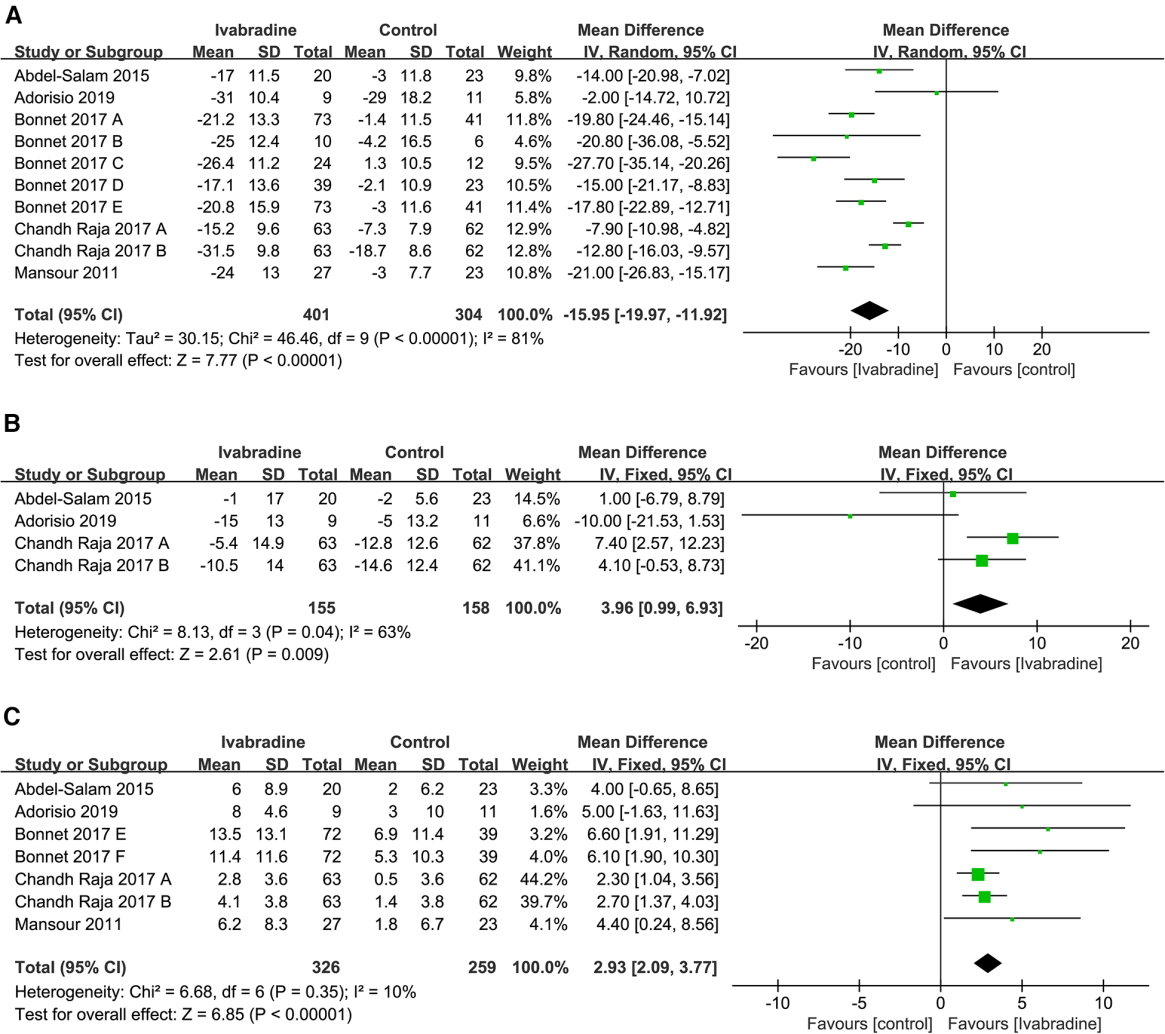
Forest plot of ivabradine intervention on RHR (**A**), SBP (**B**) and LVEF (**C**). IV, inverse variance; CI, confidence interval; RHR, resting heart rate; SBP, systolic blood pressure; LVEF, left ventricular ejection fraction.

In total, five studies involving ten comparisons reported the effect of ivabradine on RHR in patients with DCM, and outcome measures were analyzed using a random-effects model. The pooled WMD was −15.95 [95% CI (−19.97, −11.92); *I*^2^ = 81%] in Figure 3A, indicating that patients treated with ivabradine had significantly lower RHR than patients not treated with ivabradine. However, heterogeneity was evident among the different studies.

**Figure 4:**
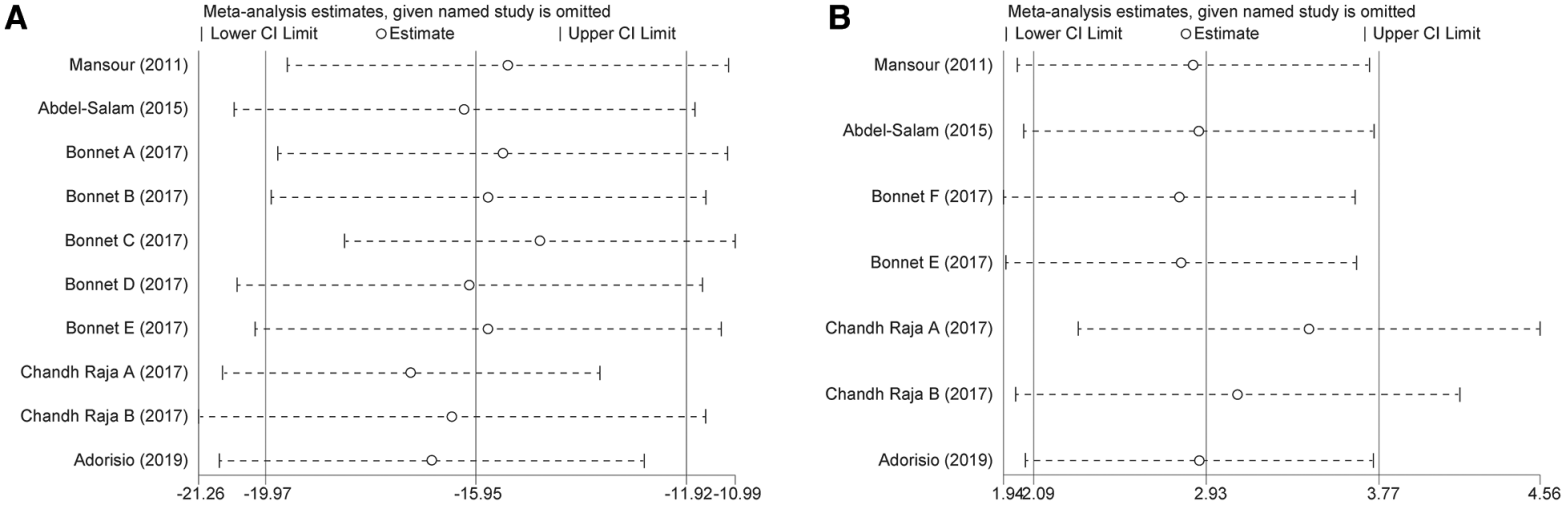
Sensitivity analysis of ivabradine intervention on RHR (**A**) and LVEF (**B**). RHR, resting heart rate; LVEF, left ventricular ejection fraction.

**Figure 5:**
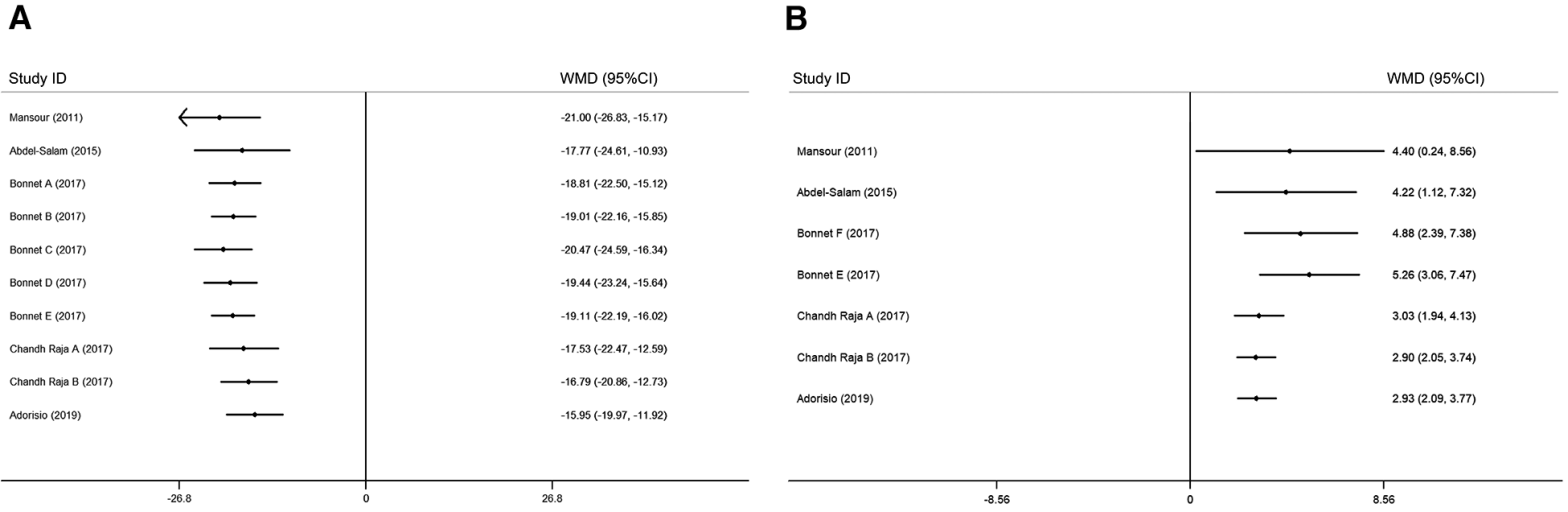
Cumulative meta-analysis according to the chronological order of publication on RHR (**A**) and LVEF (**B**). RHR, resting heart rate; LVEF, left ventricular ejection fraction.

We performed subgroup analyses to investigate whether patient age, baseline LVEF, and follow-up duration were sources of heterogeneity and whether they affected the effect of ivabradine on heart rate reduction. The results showed that the heterogeneity of the two subgroups did not decrease synchronously, indicating that these three factors did not contribute to potential heterogeneity. However, ivabradine appeared to be more effective in reducing heart rate in minor patients [WMD: −19.67; 95% CI (−23.58, −15.75) vs. WMD: −12.37; 95% CI (−17.30, −7.43)] and in patients with a higher baseline LVEF [WMD: −19.11; 95% CI (−22.19, −16.02) vs. WMD: −9.43; 95% CI (−14.07, −4.79)] (Table 2). Sensitivity analyses were performed to test the robustness of the pooled results, and the pooled effect size did not change significantly after removing each study (Figure 4A). After cumulative analysis by chronological order of publication, all aspects of the results showed no significant change trends (Figure 5A). Egger's test (*p* = 0.616) showed no publication bias (Supplementary Figure S1A).

**Table 2:** Subgroup analysis of the effects of ivabradine therapy on DCM patients.

Subgroup	RHR				LVEF			
	Studies/Patients, *n*/*N*	WMD [95% CI]	Subtotal *I*^2^	Overall *I*^2^	Studies/Patients, *n*/*N*	WMD [95% CI]	Subtotal *I*^2^	Overall *I*^2^
Age, years				81%				10%
<18	5/114	−19.67 [−23.58, −15.75]	44%		2/111	6.32 [3.20, 9.45]	0%	
≥18	5/238	−12.37 [−17.30, −7.43]	79%		5/238	2.67 [1.80, 3.54]	0%	
Follow-up, months								
<6	7/332	−17.56 [−23.30, −11.81]	85%		3/218	2.57 [1.40, 3.74]	0%	
≥6	3/259	−13.08 [−18.91, −7.25]	67%		4/256	3.31 [2.11, 4.52]	35%	
Baseline LVEF								
≤30%	3/145	−9.43 [−14.07, −4.79]	68%		3/145	2.54 [1.63, 3.44]	0%	
>30%	7/207	−19.11 [−22.19, −16.02]	38%		4/204	5.26 [3.06, 7.47]	0%	
Subgroup	LVEDV				LVESV			
	Studies/Patients, *n*/*N*	SMD [95% CI]	Subtotal *I*^2^	Overall *I*^2^	Studies/Patients, *n*/*N*	SMD [95% CI]	Subtotal *I*^2^	Overall *I*^2^
Age, years				39%				4%
<18	1/111	−0.35 [−0.74, 0.05]	–		1/111	−0.59 [−0.98, −0.19]	–	
≥18	4/218	−0.77 [−1.00, −0.54]	5%		4/218	−0.88 [−1.10, −0.66]	0%	
Follow-up, months								
<6	3/218	−0.64 [−0.92, −0.37]	0%		3/218	−0.75 [−1.02, −0.47]	0%	
≥6	2/236	−0.68 [−1.33, −0.03]	83%		2/236	−0.87 [−1.15, −0.60]	74%	
Baseline LVEF								
≤30%	2/125	−0.79 [−1.21, −0.37]	62%		2/125	−0.93 [−1.19, −0.66]	53%	
>30%	3/204	−0.53 [−0.83, −0.23]	8%		3/204	−0.67 [−0.96, −0.38]	0%	

CI, confidence interval; WMD, weighted mean differences; SMD, standardized mean differences; other abbreviations as in Table 1.

#### Systolic blood pressure

The SBP of patients with DCM was reported in three studies (19–21) involving four comparisons, and outcome measures were analyzed using a fixed effects model. The pooled WMD was only 3.96 [95% CI (0.99, 6.93); *I*^2^ = 63%] (Figure 3B), indicating that SBP was not significantly different in the ivabradine group compared to the control group. Ivabradine had little effect on SBP in patients with DCM. The heterogeneity among different studies was evident.

#### LVEF

In total, five studies involving seven comparisons reported the effect of ivabradine on LVEF in patients with DCM, and outcome measures were analyzed using a fixed effects model. The pooled WMD was 2.93 [95% CI (2.09, 3.77); *I*^2^ = 10%] (Figure 3C), indicating that patients treated with ivabradine had higher LVEF than patients not treated with ivabradine. Moreover, there was little heterogeneity among the studies.

To investigate whether patient age, baseline LVEF, and follow-up duration were sources of heterogeneity, and whether they affected the effect of ivabradine on increasing LVEF, we performed subgroup analyses. When grouped by baseline LVEF and age, there was no heterogeneity within the subgroups, suggesting that these two factors may be responsible for minor heterogeneity. It could be that minors could obtain more LVEF elevation [WMD: 6.32; 95% CI (3.20, 9.45) vs. WMD: 2.67; 95% CI (1.80, 3.54)] and patients with a higher baseline LVEF could also achieve more LVEF elevation [WMD: 5.26; 95% CI (3.06, 7.47) vs. WMD: 2.54; 95% CI (1.63, 3.44)]. However, the follow-up duration did not contribute to potential heterogeneity and was not significantly associated with the effect of ivabradine on increasing LVEF (Table 2). Sensitivity analysis was used to test the robustness of the pooled results, and the pooled effect size was slightly larger than the total pooled effect size after excluding the study by Chandh Raja (20) (Figure 4B). There was no significant change in the pooled effect size after excluding the remaining studies, and the pooled results were considered to be relatively robust. After cumulative analysis by chronological order of publication, the 95% CI narrowed, increasing the precision of estimating the overall effect size. However, the estimated value of WMD of LVEF did not show a good variation in trend, and it was significantly reduced [WMD: 5.26; 95% CI (3.06, 7.47) to WMD: 3.03; 95% CI (1.94, 4.13)] only after the last three groups (20, 21) of comparisons were added (Figure 5B).

**Figure 6:**
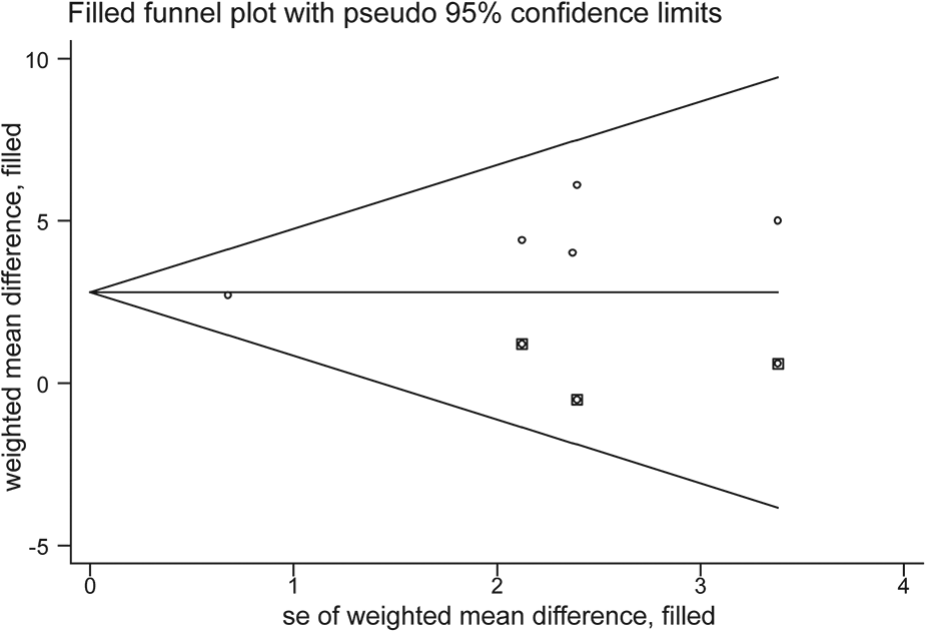
Trim-and-fill funnel plot evaluating the effect of publication bias on the pooled analysis results.

Egger's test (*p* = 0.034) revealed a significant publication bias (Supplementary Figure S1B), and it was necessary to use the trim-and-fill method to assess the stability of the pooled results. After supplementing the data of the three virtual studies (Figure 6), the pooled effect size was counted using the fixed-effect model from WMD = 3.195 to WMD = 2.798, and the *p*-value of heterogeneity test from 0.604 to 0.531. The variation in these results is minimal, indicating that the pooled analysis results were robust and unaffected by publication bias.

#### LVEDV and LVESV

**Figure 7:**
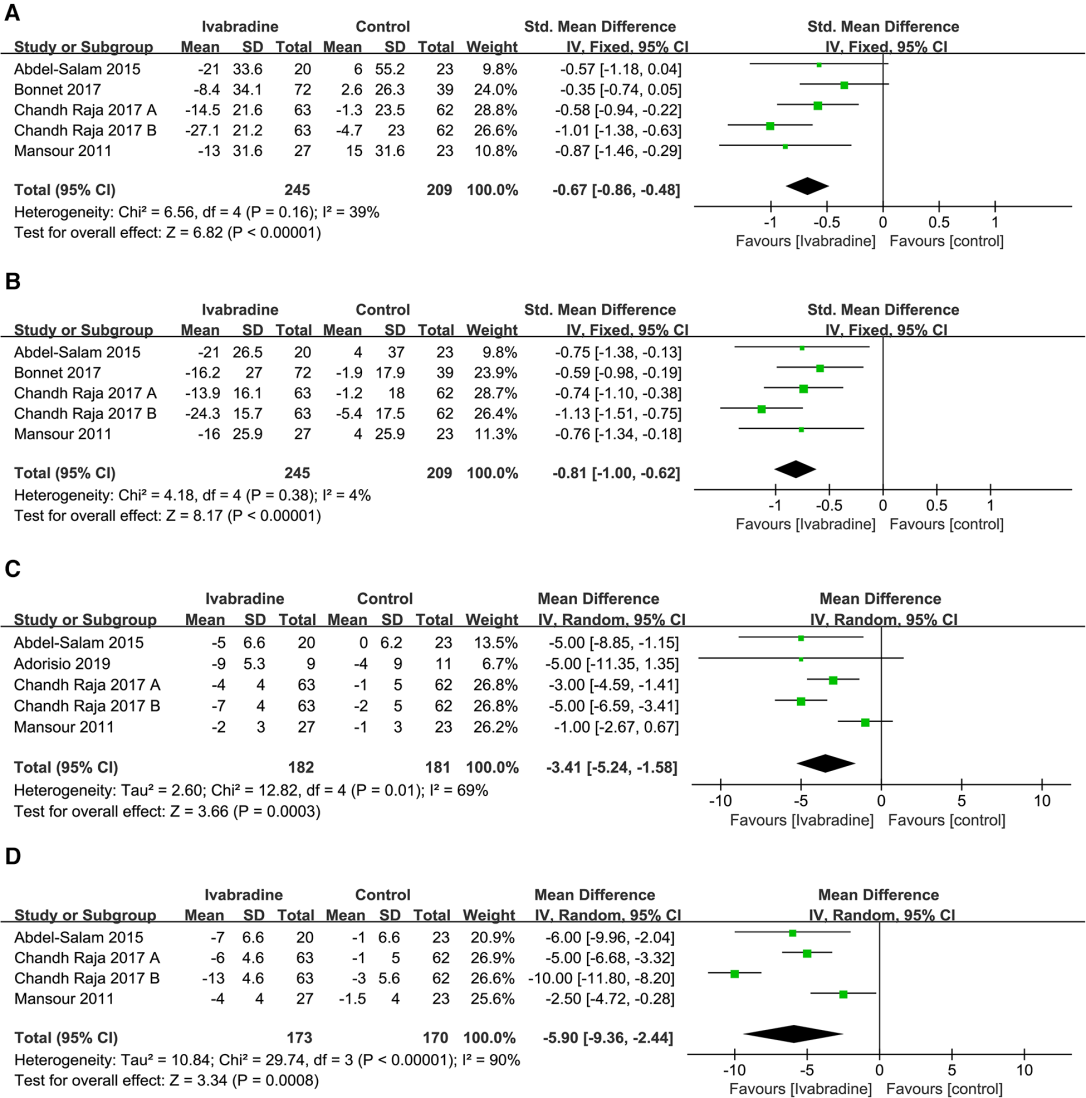
Forest plot of ivabradine intervention on LVEDV (**A**), LVESV (**B**), LVEDD (**C**) and LVESD (**D**). IV, inverse variance; CI, confidence interval; LVEDV, left ventricular end-diastolic volume; LVESV, left ventricular end-systolic volume; LVEDD, left ventricular end-diastolic diameter; LVESD, left ventricular end-systolic diameter.

In total, four studies (10, 18–20) involving five comparisons reported the effect of ivabradine on LVEDV and LVESV in patients with DCM, and outcome measures were analyzed using a fixed effects model. The pooled SMD was −0.67 [95% CI (−0.86, −0.48); *I*^2^ = 39%] (Figure 7A) and −0.81 [95% CI (−1.00, −0.62); *I*^2^ = 4%] (Figure 7B) respectively, indicating that both LVEDV and LVESV were smaller in patients treated with ivabradine than in patients not treated with ivabradine. The heterogeneity among different studies reporting both outcome measures was also low.

To investigate whether patient age, baseline LVEF, and follow-up duration were sources of heterogeneity and whether they impacted the effect of ivabradine on reducing ventricular volume, we performed subgroup analyses (Table 2). The heterogeneity of the subgroups did not decrease synchronously in the six subgroup analyses involving these two outcome measures, indicating that these three factors did not contribute to potential heterogeneity. However, adults seemed to benefit more from ivabradine in reducing ventricular volume than minors [LVEDV: SMD (adults): −0.77; 95% CI (−1.00, −0.54) vs. SMD (minors): −0.35; 95% CI (−0.74, 0.05) and LVESV: SMD (adults): −0.88; 95% CI (−1.10, −0.66) vs. SMD (minors): −0.59; 95% CI (−0.98, −0.19)].

#### LVEDD and LVESD

In total, four studies (18–21) involving 4-5 comparisons reported the effect of ivabradine on LVEDD and LVESD in patients with DCM, and the outcome measures were analyzed using a random-effects model. The pooled WMD was −3.41 [95% CI (−5.24, −1.58); *I*^2^ = 69%] (Figure 7C) and −5.90 [95% CI (−9.36, −2.24); *I*^2^ = 90%] (Figure 7D) for LVEDD and LVESD, respectively, indicating that both LVEDD and LVESD were lower in patients treated with ivabradine than in patients not treated with ivabradine. The heterogeneity between studies reporting these two outcome measures was high.

#### NYHA class

**Figure 8:**
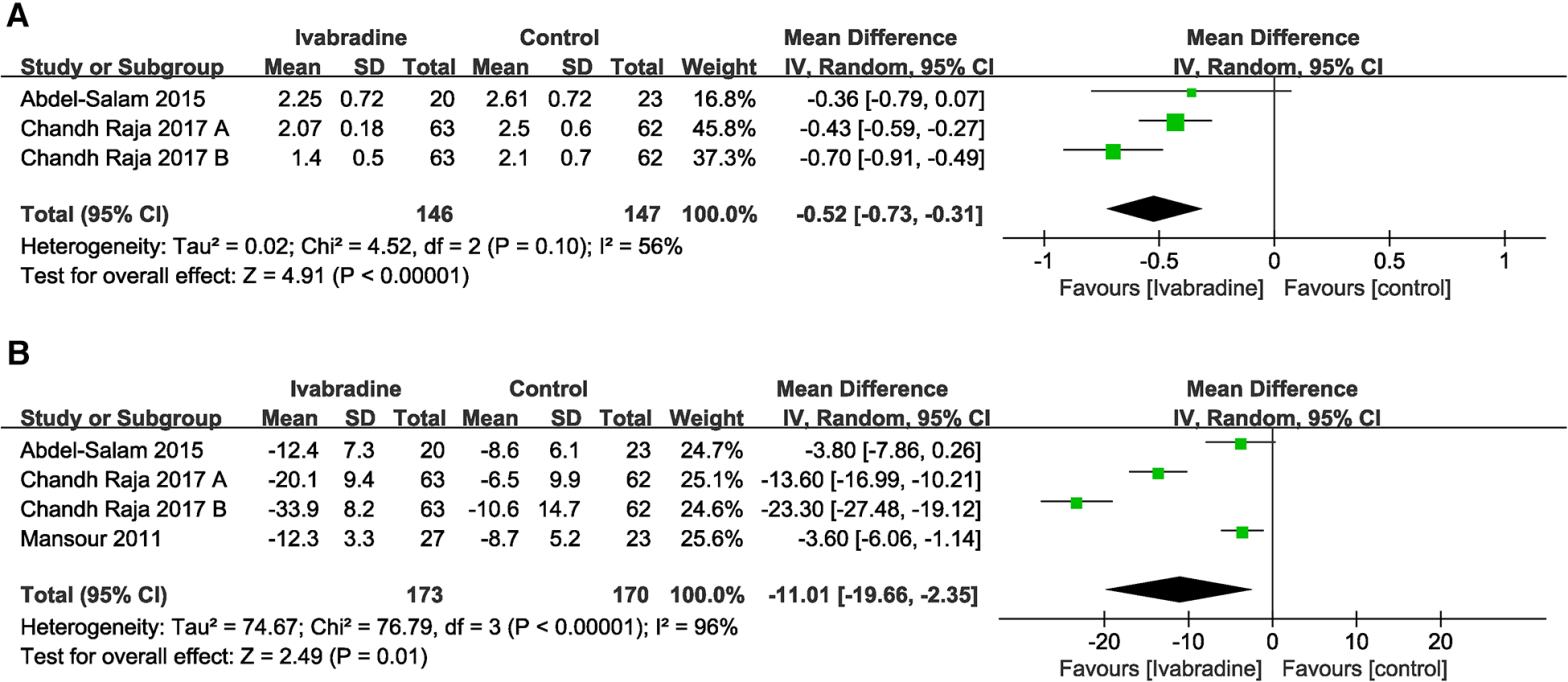
Forest plot of ivabradine intervention on NYHA class (**A**) and MLWHF score (**B**). IV, inverse variance; CI, confidence interval; NYHA class, New York heart association class; MLWHF score, Minnesota living with heart failure score.

Only two studies (19, 20) involving three comparisons reported NYHA class data, and outcome measures were analyzed using a random-effects model. The pooled WMD was −0.52 [95% CI (−0.73, −0.31); *I*^2 ^= 56%] (Figure 8A), indicating that NYHA class in the ivabradine group was significantly improved compared with the control group. There was relatively obvious heterogeneity among the different studies.

#### MLWHF score

In total, three studies (18–20) involving four comparisons evaluated the MLWHF scores of patients with DCM, and outcome measures were analyzed using a random-effects model. The pooled WMD was −11.01 [95% CI (−19.66, −2.35); *I*^2 ^= 96%] (Figure 8B), indicating that the MLWHF score in the ivabradine group was significantly improved compared with that in the control group, and there was obvious heterogeneity among different studies.

#### All-cause mortality and cardiac mortality

**Figure 9:**
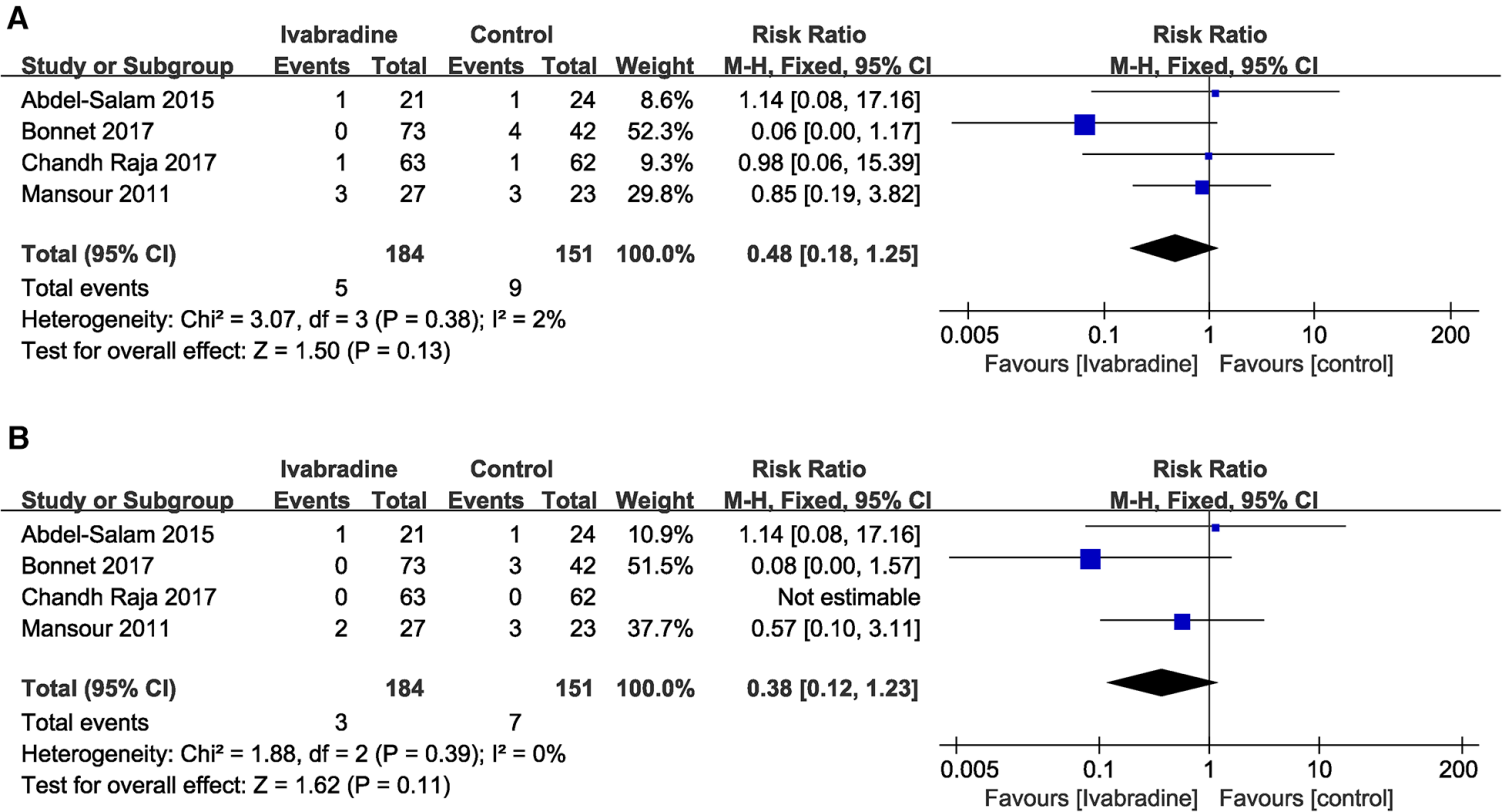
Forest plot of ivabradine intervention on all-cause mortality (**A**) and cardiac mortality (**B**). M-H, mantel-haenzel; CI, confidence interval.

In total, four (10, 18–20) studies reported the effect of ivabradine on all-cause and cardiac mortality in patients with DCM, and we used the Mantel-Haenszel method and a fixed effects model for analysis. The pooled RR was 0.48 [95% CI (0.18, 1.25); *I*^2^ = 2%] (Figure 9A) and 0.38 [95% CI (0.12, 1.23); *I*^2 ^= 0%] respectively (Figure 9B) and there was little heterogeneity among the studies. Notably, in Bonnet's study (10) (the patients included were all minors), the RR of both outcome measures was significantly lower than that in other studies. However, the pooled RR for both all-cause mortality (*p* = 0.13) and cardiac mortality (*p* = 0.11) was not statistically significant.

### Quality of the evidence

The quality of evidence was very low, low or moderate for all outcome measures (Supplementary Table S2). Among the five studies included, there are some concerns about overall risk of bias in four of them, with one exhibiting a high overall risk of bias. Therefore, the quality of evidence was downgraded in view of study limitations. The continuous and dichotomous outcome measures were then downgraded as a result of the imprecision of the small sample sizes and wide CIs (including both null effect and appreciable benefit or harm), respectively. The quality of evidence for SBP was downgraded due to inconsistency, as the direction of the results from various studies differed, and significant heterogeneity was present. Because the RR of all-cause and cardiac mortality were low, we upgraded the quality of evidence. The criteria for downgrading were not fully met in terms of indirectness and publication bias; therefore, downgrading was not considered.

## Discussion

Through statistical analysis of the five included trials, we found that ivabradine significantly reduced heart rate and partly improved cardiac function (including LVEF, ventricular volume, and NYHA class) and quality of life, but had no significant effect on SBP and prognosis (all-cause mortality and cardiac mortality) in patients with DCM.

At present, there is no systematic review focusing on the treatment of DCM with ivabradine; however, in the past, there have been many clinical studies and systematic reviews reporting its effectiveness in the treatment of HF, which is one of the main clinical features of DCM. Many SHIFT trials (Systolic HF Treatment with the I_f_ Inhibitor Ivabradine Trial) support the conclusion that ivabradine improves HF-related quality of life, clinical outcomes of HF, and cardiac function (22–24), and these effects are closely related to its effect on reducing heart rate. Ivabradine can selectively block hyperpolarization-activated cyclic nucleotide–gated (HCN) channels, which are involved in the formation of the I_f_ current in the early phase 4 spontaneous depolarization of autorhythmic cells. When the I_f_ current of sinoatrial node cells weakens, the rate of phase 4 spontaneous depolarization slows, which reduces the autorhythmicity of the sinoatrial node and further reduces the heart rate (25). The relationship between heart rate and patient prognosis has long been confirmed by clinical studies (26). In addition, several clinical studies have reported that reductions in heart rate are associated with improvements in health-related quality of life and reductions in the risk of developing adverse cardiovascular outcomes (23, 27); a rapid heart rate leads to reductions in LVEF, peak oxygen consumption, and 6-minute walk distance (28) as well. The three studies we included also reported the relationship between lowering heart rate in patients with DCM and improvement in certain outcome measures. However, the pathophysiological mechanism underlying the relationship between heart rate and HF development is still unclear. Two studies provided explanations: the reduction in heart rate increases ventricular blood flow and oxygen uptake by increasing diastolic duration, thereby promoting myocardial energy metabolism (29); slowing down heart rate can reverse the adverse changes in excitation-contraction coupling of heart failure (30). Furthermore, several studies have found that ivabradine can improve cardiac function through alternative mechanisms: ivabradine reduces adverse remodeling and injury in the heart by inhibiting calcium overload induced by increased I_f_ current (31). Shuai et al. (32) found that ivabradine ameliorated pressure overload-induced myocardial fibrosis and cardiac dysfunction by upregulating miR-133a in mice. Ivabradine may mediate the immunomodulatory and anti-inflammatory effects independent of heart rate reduction (33, 34).

Although there have been many literature reports on the treatment of HF with ivabradine, DCM often manifests as various arrhythmias in addition to CHF. Arrhythmias can occur at any stage of the DCM and has a higher incidence than at the course of chronic CHF caused by other common reasons (28). Ventricular tachyarrhythmia and high-grade atrioventricular block are the main causes of sudden death in patients with DCM (35). With the improvement of standardized treatment for HF, the occurrence of various malignant arrhythmias may have a more important impact on the prognosis of patients with DCM than HF (36). Animal experiments have shown that ivabradine can significantly reduce the incidence, duration, and arrhythmia-related mortality of ventricular arrhythmia in animals with myocardial infarction and HF (37, 38). Currently, the most widely recognized mechanism is that HCN channels expression is abnormally increased in the ventricles during ventricular dilatation, myocardial infarction, and HF, and the enhancement of I_f_ current causes increased ventricular autorhythmicity, which leads to the occurrence of ventricular tachyarrhythmia (39). Ivabradine exerts its therapeutic effect on arrhythmia by blocking HCN channels and inhibiting its overexpression (37–40). We therefore suspect that ivabradine is more effective in reducing mortality in DCM patients than in all-cause HF. Several clinical trials have not shown a significant effect of Ivabradine on sudden cardiac death in patients with chronic heart failure (24, 41), and systematic reviews that included these studies also yielded negative results (42, 43). The reason may be that the incidence of malignant arrhythmias is particularly low, and β-blockers as background therapy can already reduce the incidence of arrhythmias (24, 41). However, our study also did not demonstrate any beneficial effect of ivabradine on the prognosis of patients. So it doesn't validate our previous conjecture. Regarding the two prognostic measures, the four included studies involved only a small number of patients, so reliable and generalizable conclusions cannot be drawn. We hope that subsequent studies will further explore this issue.

Our subgroup analysis led to some interesting conclusions: (1) Ivabradine is more effective in reducing heart rate in juvenile patients, as well as in patients with a higher baseline LVEF; (2) Juvenile patients and patients with higher baseline LVEF can obtain more LVEF improvement. We believe that, although as mentioned earlier, other factors may play a potential role, heart rate reduction is the primary reason for ivabradine's improvement of cardiac function. The heart rate of minors, especially children aged 0–3 years, is significantly higher than that of adults (44), and the higher basal heart rate may be responsible for the better efficacy of ivabradine. A higher baseline LVEF may indicates a less severe degree of cardiac dysfunction and a relatively milder influence of various neurohumoral regulatory mechanisms (45); therefore, it is possible that the heart is more responsive to ivabradine and thus exhibits a higher potential for improving cardiac function. However, the above explanations for the results of the subgroup analysis do not have much literature basis, and the validity of these conclusions needs to be further confirmed by subsequent studies. The results of the cumulative analysis only showed that the estimates of efficacy for improving LVEF in patients with DCM became more precise as ivabradine was investigated over the last 10 years.

Our systematic review explores the effectiveness and possible mechanisms of ivabradine in the treatment of DCM. And also compared prognostic outcome measures with those from previous SHIFT trials and corresponding systematic reviews in the expectation of finding uniqueness of ivabradine in the treatment of DCM, although no meaningful conclusions can be drawn due to negative results. In general, this study provides some guidance for the clinical use of ivabradine to some extent.

## Limitations

This study had several limitations. First, the small number of studies included not only reduced the validity of the pooled statistics but also limited subsequent subgroup and cumulative analyses. Second, objective statistical sample size of prognosis outcome measures is limited; therefore, larger and better-designed randomized controlled trials are needed to determine the prognostic impact of ivabradine on patients. Third, both the risk of bias in our included studies and the quality of the evidence on the outcome measures were less than satisfactory, which somewhat reduced the credibility of our conclusions. Fourth, there was significant heterogeneity across the included studies in WMD for RHR, left ventricular diameter, NYHA class, and MLWHF score. Although sensitivity and subgroup analyses were used, the source of heterogeneity could not be completely identified. Fifth, in the statistical analysis of LVEF we found that each comparison accounted for a significantly uneven weight. If the study quality and publication bias of the two comparisons (20) accounting for 83.9% were poor, it would have a great impact on the overall effect size. Sixth, Egger's test has a relatively lower power when the number of studies included in the meta-analysis is less than 10. Hence, publication bias may not be detected by Egger's test. Seventh, this study protocol was not registered in an openly accessible database, which could reduce the transparency and credibility of the study and increase the risk of duplication of research.

## Conclusions

Our systematic review suggests that ivabradine can reduce heart rate and ventricular volume, and improve cardiac function in patients with DCM. Moreover, it may be more effective in juvenile patients and patients with higher baseline LVEF. However, more high-quality clinical studies are still needed to explore the efficacy of ivabradine in DCM.

## Data Availability

The original contributions presented in the study are included in the article/**Supplementary Material**, further inquiries can be directed to the corresponding authors.
