# Supplementary material for: The effect of ivabradine therapy on dilated cardiomyopathy patients with congestive heart failure: a systematic review and meta-analysis
Source: Front Cardiovasc Med. 2023 Oct 17;10:1149351. doi: 10.3389/fcvm.2023.1149351 (PMC10616249; doi:10.3389/fcvm.2023.1149351)
Supplement: Supplementary file 1 [file Datasheet1.pdf]

Supplementary Material

Item No	Title
Supplementary Table S1	GRADE Grading Summary Table
Supplementary Table S2	Summary of findings for all included outcome measures
Supplementary Table S3	PRISMA 2020 Main Checklist
Supplementary Table S4	PRISMA Abstract Checklist
Supplementary Figure S1	Funnel plot of Egger’s test
Supplementary Methods	Search sequences that were used in the different databases

This supplementary material has been provided by the authors to give readers additional information about their work.

Supplementary Table S1. GRADE Grading Summary Table

Maximum deduction score	Downgrade factors	Downgrade criteria		Operating rules
		GRADE	Team interpretation	
-1	Publication bias	1. Empirical evidence indicates that studies with statistically significant results are more likely to be published compared to studies with non-significant results (i.e., negative result studies). In recent years, some revelations have shown that the concealment of "negative" results from industry-sponsored research is quite common.<br>2. When only a small number of preliminary studies are available early on, completed systematic reviews tend to overestimate the effect size, particularly when "negative result" studies are published with a delay.<br>3. Early positive studies with small sample sizes, especially small-scale trials, are worthy of skepticism.<br>4. Systematic review authors should pay particular attention to publication bias, especially when the included primary studies have small sample sizes, and particularly when small-scale studies are funded by industry.<br>5. Classic methods for examining the type of test results (e.g., funnel plots) may indicate publication bias but require careful interpretation.	1. All included RCT studies had small sample sizes (30 pairs), especially those sponsored by manufacturers.<br>2. An asymmetric funnel plot or statistical tests indicating the presence of publication bias can significantly influence the results.	Meet both criteria, -1
-2	Imprecision	1. Confidence intervals that cross the clinical decision thresholds for recommending or not recommending treatment may lead to downgrading due to imprecision.<br>2. If the confidence intervals do not cross over, does the study meet the OIS criteria? Alternatively, is the event rate very low with a large sample size (at least 2000, possibly 4000 cases)? If it fails to meet either of these criteria, the evidence quality level may be downgraded due to imprecision.	1. The 95% confidence interval (CI) crosses the line (MD only considers the equivalence line).<br>(1) Equivalence line: 1 or 0<br>(2) Clinically significant benefit or harm: RR value of 0.75 or 1.25<br>2. The total number of events or cases does not exceed OIS (the total sample size including all studies, <300 for categorical variables, and <400 for continuous variables).	Meet one of the criteria, -1<br>Meet two of the criteria, -2
-2	Study limitations	1. Lack of allocation concealment: The individuals recruiting the participants are aware of the group (or phase in a crossover trial) to which the next participant will be assigned (the main issue in "fake" or "semi" randomization trials, where allocation is based on factors like the day of the week, birth date, or chart number).<br>2. Lack of blinding: Patients, caregivers, outcome assessors, adjudicators, or data analysts are aware of the group to which the patients are assigned (or the current drug treatment they are receiving in a	Based on the COCHRANE bias risk assessment results, deducting points conservatively.<br>1. All or most (2/3) information comes from low risk.<br>2. Most (2/3) information comes from some risk.<br>3. Most (2/3) information comes from high risk.	1. no deduction<br>2. minus one point<br>3. minus one point

		crossover trial).<br>3. Incomplete reporting of patients and outcome events: Loss to follow-up and non-adherence to intention-to-treat principles in superiority trials; or loss to follow-up and non-simultaneous conduct of two analyses in non-inferiority trials: one analyzing only patients who adhered to the treatment and the other analyzing all available outcome data.<br>4. Selective outcome reporting bias: Incomplete reporting or non-disclosure of certain results and other outcome-related information.<br>5. Other limitations: Trial termination due to early benefit; use of unvalidated outcome measurement methods (e.g., patient-reported outcomes); carryover effects in crossover trials; recruitment bias in cluster randomized trials.		
-2	Inconsistency	1. The point estimates vary widely among different studies.<br>2. The confidence intervals are very narrow or do not overlap.<br>3. Heterogeneity test - testing the null hypothesis that the effect sizes of all studies included in the meta-analysis are the same - yields a very small p-value.<br>4. I^2 - quantifies the proportion of variation in the point estimates that is due to heterogeneity among studies - has a high value.	Based on I^2, if I^2 is large and cannot be explained, it may be considered to downgrade the quality; if the heterogeneity is primarily due to differences in effect size (high heterogeneity, but the direction is the same), and it does not affect the conclusion, considering not downgrading may be appropriate.<br>1. The point estimates vary widely among different studies.<br>2. The confidence intervals are very narrow or do not overlap.<br>3. The p-value from the heterogeneity test is very small.<br>4. The I^2 value from the heterogeneity test is large (75%).	If the direction of the results is different, refer to the following criteria:<br>$50\%<I^2<75\%$, -1<br>$I^2\geq75\%$, -2
-2	Indirectness	1. Population differences (applicability);<br>2. Intervention differences (applicability);<br>3. Outcome measurement differences (surrogate outcomes);<br>4. Indirect comparison.	1. Population differences (animal/human, adults/children, hospital/community, etc.).<br>2. Intervention differences (dosage, timing, etc.).<br>3. Outcome measurement differences (surrogate endpoints). For example, an indirect source of evidence related to outcome measurement is the use of surrogate or substitute endpoints to replace the clinically relevant patient-important outcomes of interest.<br>4. Indirect comparison (network meta-analysis).	Virtually no consideration of downgrading

Abbreviation: RCT = randomized controlled trial; OIS = optimal information size; MD = mean difference; RR = risk ratio.

Supplementary Table S2. Summary of findings for all included outcome measures

Ivabradine compared to Control for improving prognosis and cardiac function of patients with dilated cardiomyopathy

Patient or population: patients were diagnosed with DCM with congestive heart failure

Settings: hospital

Intervention: Ivabradine

Comparison: Control

Outcomes	Illustrative comparative risks* (95% CI)		Relative effect (95% CI)	No of Participants (studies)	Quality of the evidence (GRADE)	Comments
	Assumed risk<br>**Control**	Corresponding risk<br>**Ivabradine**				
**RHR**	The mean RHR in the control groups was -**7.69 bpm**	The mean RHR in the intervention groups was **15.95 lower** (19.97 to 11.92 lower)	-	352<br>(10 studies)	⊕⊕⊖⊖<br>**low**[1,2]	Ivabradine provides significant benefit.
**LVEF**<br>Follow-up: mean 6 months	The mean LVEF in the control groups was **2.76 %**	The mean LVEF in the intervention groups was **2.93 higher** (2.09 to 3.77 higher)	-	349<br>(7 studies)	⊕⊕⊖⊖<br>**low**[1,2]	Ivabradine provides some benefit.
**MLWHF score**<br>Scale from: 0 to 105, 0 is best.<br>Follow-up: mean 4 months	The mean MLWHF score in the control groups was **-8.58**	The mean MLWHF score in the intervention groups was **11.01 lower** (19.66 to 2.35 lower)	-	218<br>(4 studies)	⊕⊕⊖⊖<br>**low**[1,2]	Ivabradine provides some benefit.
**LVEDV**<br>Follow-up: mean 6 months	The mean LVEDV in the control groups was **1.02 ml**	The mean LVEDV in the intervention groups was **0.67 standard deviations lower** (0.86 to 0.48 lower)	-	329<br>(5 studies)	⊕⊕⊖⊖<br>**low**[1,2]	The efficacy of ivabradine is difficult to evaluate.<br>SMD -0.67 (-0.86 to -0.48)
**LVESV**<br>Follow-up: mean 6 months	The mean LVESV in the control groups was **-1.43 ml**	The mean LVESV in the intervention groups was **0.81 standard deviations lower** (1 to 0.62 lower)	-	329<br>(5 studies)	⊕⊕⊖⊖<br>**low**[1,2]	The efficacy of ivabradine is difficult to evaluate.<br>SMD -0.81 (-1 to -0.62)
**LVEDD**<br>Follow-up: mean 4.5 months	The mean LVEDD in the control groups was **-1.40 mm**	The mean LVEDD in the intervention groups was **3.41 lower** (5.24 to 1.58 lower)	-	238<br>(5 studies)	⊕⊕⊖⊖<br>**low**[1,2]	Ivabradine provides some benefit.
**LVESD**<br>Follow-up: mean 4 months	The mean LVESD in the control groups was **-1.80 mm**	The mean LVESD in the intervention groups was **5.9 lower** (9.36 to 2.44 lower)	-	218<br>(4 studies)	⊕⊕⊖⊖<br>**low**[1,2]	Ivabradine provides some benefit.

**SBP**<br>Follow-up: mean 5 months	The mean SBP in the control groups was **-11.39 mmHg**	The mean SBP in the intervention groups was 3.96 **higher** (0.99 to 6.93 higher)	-	188<br>(4 studies)	⊕⊖⊖⊖<br>**very low**[1,2,3]	Ivabradine may have little or no effect.
**NYHA class**<br>Scale from: I to IV, I is best.<br>Follow-up: mean 4 months	The mean NYHA class in the control groups was **2.35**	The mean NYHA class in the intervention groups was **0.52 lower** (0.73 to 0.31 lower)	-	168<br>(3 studies)	⊕⊕⊖⊖<br>**low**[1,2]	Ivabradine provides significant benefit.
**All-cause mortality**<br>Follow-up: mean 9 months	**60 per 1000**	**29 per 1000**<br>(11 to 75)	**RR 0.48**<br>(0.18 to 1.25)	335<br>(4 studies)	⊕⊕⊕⊖<br>**moderate**[1,4,5]	The result of the pooled analysis is negative.
**Cardiac mortality**<br>Follow-up: mean 9 months	**46 per 1000**	**18 per 1000**<br>(6 to 57)	**RR 0.38**<br>(0.12 to 1.23)	335<br>(4 studies)	⊕⊕⊕⊖<br>**moderate**[1,4,5]	The result of the pooled analysis is negative.

*The basis for the **assumed risk** (e.g. the median control group risk across studies) is provided in footnotes. The **corresponding risk** (and its 95% confidence interval) is based on the assumed risk in the comparison group and the **relative effect** of the intervention (and its 95% CI).

CI: Confidence interval; **RR:** Risk ratio;

GRADE Working Group grades of evidence
High quality: Further research is very unlikely to change our confidence in the estimate of effect.
Moderate quality: Further research is likely to have an important impact on our confidence in the estimate of effect and may change the estimate.
Low quality: Further research is very likely to have an important impact on our confidence in the estimate of effect and is likely to change the estimate.
Very low quality: We are very uncertain about the estimate.

[1] Downgraded one level due to study limitations, as most of the information comes from studies with moderate risk of bias, and one study reported high risk of bias for RHR, LVEF, LVEDD, and SBP.
[2] Downgraded one level due to imprecision, as the sample size included in the study was small.
[3] Downgraded one level due to inconsistency, as I^2 is between 50% and 75%, and the direction of the results is inconsistent.
[4] Downgraded one level due to imprecision, as the confidence interval is wide.
[5] Upgraded one level due to large effect, as the RR is lower than 0.5.

Abbreviation: DCM = dilated cardiomyopathy; CI = confidence interval; RHR = resting heart rate; bpm = beats per minute; LVEF = Left ventricular ejection fraction; MLWHF score = Minnesota Living with Heart Failure score; LVEDV = left ventricular end-diastolic volume; SMD = standard mean difference; LVESV = left ventricular end-systolic volume; LVEDD = left ventricular end-diastolic diameter; LVESD = left ventricular end-systolic diameter; SBP = systolic blood pressure; NYHA class = New York Heart Association class; RR = risk ratio.

Supplementary Table S3. PRISMA 2020 Main Checklist

Topic	No.	Item	Location where item is reported
**TITLE**			

Topic	No.	Item	Location where item is reported
**Title**	1	Identify the report as a systematic review.	1
**ABSTRACT**			
**Abstract**	2	See the PRISMA 2020 for Abstracts checklist	
**INTRODUCTION**			
**Rationale**	3	Describe the rationale for the review in the context of existing knowledge.	2-3
**Objectives**	4	Provide an explicit statement of the objective(s) or question(s) the review addresses.	3
**METHODS**			
**Eligibility criteria**	5	Specify the inclusion and exclusion criteria for the review and how studies were grouped for the syntheses.	4
**Information sources**	6	Specify all databases, registers, websites, organisations, reference lists and other sources searched or consulted to identify studies. Specify the date when each source was last searched or consulted.	3
**Search strategy**	7	Present the full search strategies for all databases, registers and websites, including any filters and limits used.	3, supplementary methods
**Selection process**	8	Specify the methods used to decide whether a study met the inclusion criteria of the review, including how many reviewers screened each record and each report retrieved, whether they worked independently, and if applicable, details of automation tools used in the process.	3
**Data collection process**	9	Specify the methods used to collect data from reports, including how many reviewers collected data from each report, whether they worked independently, any processes for obtaining or confirming data from study investigators, and if applicable, details of automation tools used in the process.	4
**Data items**	10a	List and define all outcomes for which data were sought. Specify whether all results that were compatible with each outcome domain in each study were sought (e.g. for all measures, time points, analyses), and if not, the methods used to decide which results to collect.	4
	10b	List and define all other variables for which data were sought (e.g. participant and intervention characteristics, funding sources). Describe any assumptions made about any missing or unclear information.	4
**Study risk of bias assessment**	11	Specify the methods used to assess risk of bias in the included studies, including details of the tool(s) used, how many reviewers assessed each study and whether they worked independently, and if applicable, details of automation tools used in the process.	4
**Effect measures**	12	Specify for each outcome the effect measure(s) (e.g. risk ratio, mean difference) used in the synthesis or presentation of results.	4-5
**Synthesis methods**	13a	Describe the processes used to decide which studies were eligible for each synthesis (e.g. tabulating the study intervention characteristics and comparing against the planned groups for each synthesis (item 5)).	4
	13b	Describe any methods required to prepare the data for presentation or synthesis, such as handling of missing summary statistics, or data conversions.	5
	13c	Describe any methods used to tabulate or visually display results of individual studies and syntheses.	4

Topic	No.	Item	Location where item is reported
	13d	Describe any methods used to synthesize results and provide a rationale for the choice(s). If meta-analysis was performed, describe the model(s), method(s) to identify the presence and extent of statistical heterogeneity, and software package(s) used.	5
	13e	Describe any methods used to explore possible causes of heterogeneity among study results (e.g. subgroup analysis, meta-regression).	5
	13f	Describe any sensitivity analyses conducted to assess robustness of the synthesized results.	5
**Reporting bias assessment**	14	Describe any methods used to assess risk of bias due to missing results in a synthesis (arising from reporting biases).	5
**Certainty assessment**	15	Describe any methods used to assess certainty (or confidence) in the body of evidence for an outcome.	5, Supplementary Table 1
**RESULTS**			
**Study selection**	16a	Describe the results of the search and selection process, from the number of records identified in the search to the number of studies included in the review, ideally using a flow diagram.	6, Figure 1
	16b	Cite studies that might appear to meet the inclusion criteria, but which were excluded, and explain why they were excluded.	6, Figure 1
**Study characteristics**	17	Cite each included study and present its characteristics.	6, Table 1
**Risk of bias in studies**	18	Present assessments of risk of bias for each included study.	6, Figure 2
**Results of individual studies**	19	For all outcomes, present, for each study: (a) summary statistics for each group (where appropriate) and (b) an effect estimate and its precision (e.g. confidence/credible interval), ideally using structured tables or plots.	6-9, Figure 3,7-9
**Results of syntheses**	20a	For each synthesis, briefly summarise the characteristics and risk of bias among contributing studies.	6
	20b	Present results of all statistical syntheses conducted. If meta-analysis was done, present for each the summary estimate and its precision (e.g. confidence/credible interval) and measures of statistical heterogeneity. If comparing groups, describe the direction of the effect.	7-9, Figure 3,7-9
	20c	Present results of all investigations of possible causes of heterogeneity among study results.	7-8, Table 2
	20d	Present results of all sensitivity analyses conducted to assess the robustness of the synthesized results.	7, Figure 4
**Reporting biases**	21	Present assessments of risk of bias due to missing results (arising from reporting biases) for each synthesis assessed.	7-8, Figure 6, Supplementary Figure 1
**Certainty of evidence**	22	Present assessments of certainty (or confidence) in the body of evidence for each outcome assessed.	9, Supplementary Table 2
**DISCUSSION**			
**Discussion**	23a	Provide a general interpretation of the results in the context of other evidence.	9-11
	23b	Discuss any limitations of the evidence included in the review.	11
	23c	Discuss any limitations of the review processes used.	11-12
	23d	Discuss implications of the results for practice, policy, and future research.	11
**OTHER INFORMATION**			

Topic	No.	Item	Location where item is reported
**Registration and protocol**	24a	Provide registration information for the review, including register name and registration number, or state that the review was not registered.	13
	24b	Indicate where the review protocol can be accessed, or state that a protocol was not prepared.	13
	24c	Describe and explain any amendments to information provided at registration or in the protocol.	-
**Support**	25	Describe sources of financial or non-financial support for the review, and the role of the funders or sponsors in the review.	12
**Competing interests**	26	Declare any competing interests of review authors.	13
**Availability of data, code and other materials**	27	Report which of the following are publicly available and where they can be found: template data collection forms; data extracted from included studies; data used for all analyses; analytic code; any other materials used in the review.	12

Supplementary Table S4. PRISMA Abstract Checklist

Topic	No.	Item	Reported?
**TITLE**			
**Title**	1	Identify the report as a systematic review.	No
**BACKGROUND**			
**Objectives**	2	Provide an explicit statement of the main objective(s) or question(s) the review addresses.	Yes
**METHODS**			
**Eligibility criteria**	3	Specify the inclusion and exclusion criteria for the review.	Yes
**Information sources**	4	Specify the information sources (e.g. databases, registers) used to identify studies and the date when each was last searched.	Yes
**Risk of bias**	5	Specify the methods used to assess risk of bias in the included studies.	Yes
**Synthesis of results**	6	Specify the methods used to present and synthesize results.	Yes
**RESULTS**			
**Included studies**	7	Give the total number of included studies and participants and summarise relevant characteristics of studies.	Yes
**Synthesis of results**	8	Present results for main outcomes, preferably indicating the number of included studies and participants for each. If meta-analysis was done, report the summary estimate and confidence/credible interval. If comparing groups, indicate the direction of the effect (i.e. which group is favoured).	Yes
**DISCUSSION**			
**Limitations of evidence**	9	Provide a brief summary of the limitations of the evidence included in the review (e.g. study risk of bias, inconsistency and imprecision).	Yes
**Interpretation**	10	Provide a general interpretation of the results and important implications.	Yes
**OTHER**			
**Funding**	11	Specify the primary source of funding for the review.	No
**Registration**	12	Provide the register name and registration number.	No

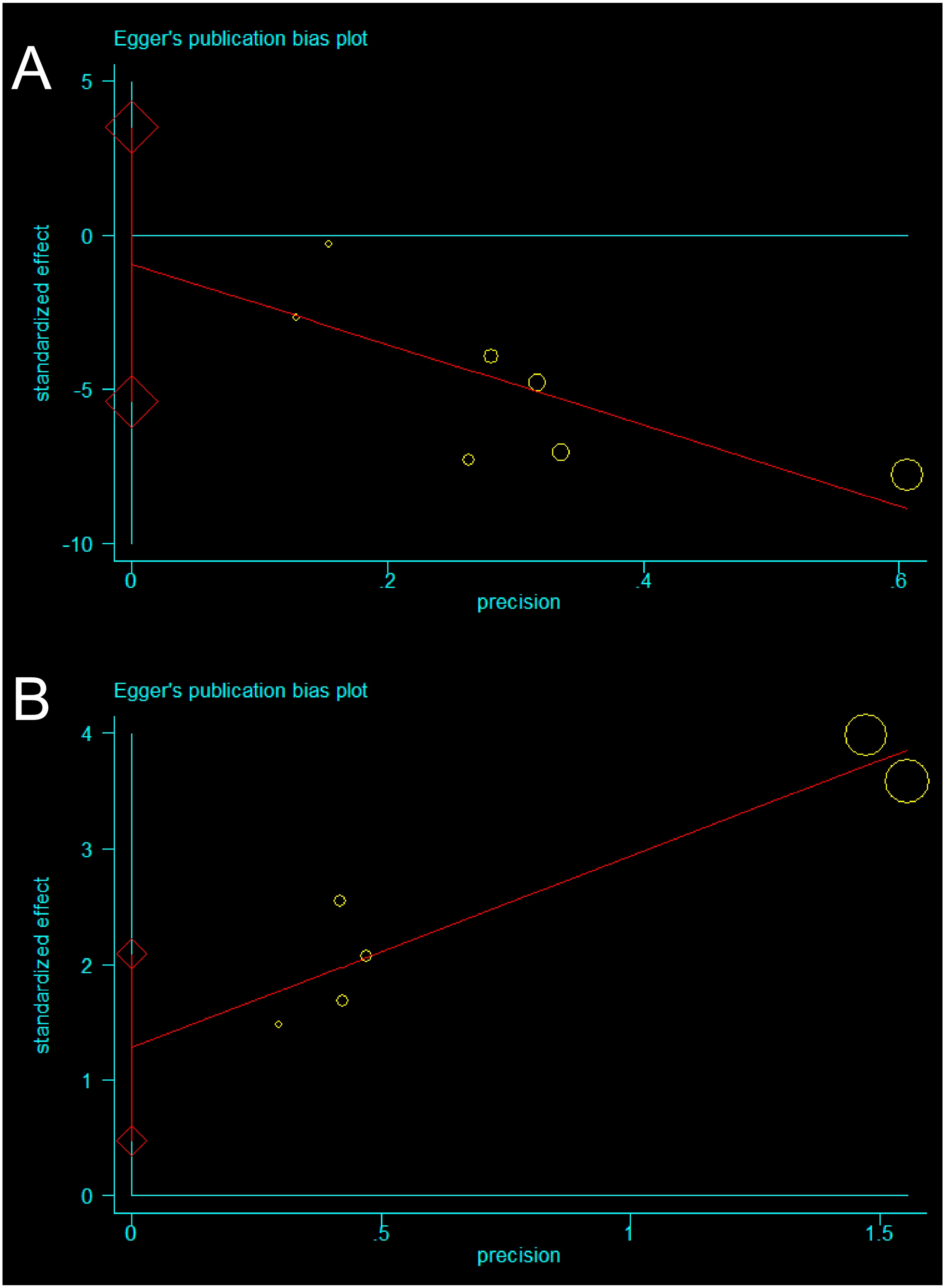

Supplementary Figure S1. Funnel plot of Egger's test with (A) rest heart rate, (B) left ventricular ejection fraction.

Supplementary Methods: Search sequences that were used in the different databases

- PubMed:

((Ivabradine[Title/Abstract]) OR (Corlanor[Title/Abstract])) AND ((((((Cardiomyopathy, Dilated[Title/Abstract]) OR (Dilated Cardiomyopathies[Title/Abstract])) OR (Dilated Cardiomyopathy[Title/Abstract])) OR (Familial Idiopathic Cardiomyopathy[Title/Abstract])) OR (Congestive Cardiomyopathy[Title/Abstract])) OR (Congestive Cardiomyopathies[Title/Abstract]))

- Embase

Search #3

#3. #1 AND #2

#2. ivabradine:ab,ti OR corlanor:ab,ti

#1. 'cardiomyopathy, dilated':ab,ti OR 'dilated cardiomyopathies':ab,ti OR 'dilated cardiomyopathy':ab,ti OR 'familial idiopathic cardiomyopathy':ab,ti OR 'congestive cardiomyopathy':ab,ti OR 'congestive cardiomyopathies':ab,ti

- Web of science

Search 3

1: (((((TS=(Cardiomyopathy, Dilated)) OR TS=(Dilated Cardiomyopathies)) OR TS=(Dilated Cardiomyopathy)) OR TS=(Familial Idiopathic Cardiomyopathy)) OR TS=(Congestive Cardiomyopathy)) OR TS=(Congestive Cardiomyopathies)

2: (TS=(Ivabradine)) OR TS=(Corlanor)

3: #2 AND #1

- Cochrane library

Search #5

#1 MeSH descriptor: [Cardiomyopathy, Dilated] explode all trees

#2 (Dilated Cardiomyopathies):ti,ab,kw OR (Dilated Cardiomyopathy):ti,ab,kw OR (Familial Idiopathic Cardiomyopathy):ti,ab,kw OR (Congestive Cardiomyopathy):ti,ab,kw OR (Cardiomyopathy, Dilated):ti,ab,kw (Word variations have been searched)

#3 #1 OR #2

#4 (ivabradine):ti,ab,kw OR (Corlanor):ti,ab,kw (Word variations have been searched)

#5 #3 AND #4
