# Supplementary figures and images for: The effect of ivabradine therapy on dilated cardiomyopathy patients with congestive heart failure: a systematic review and meta-analysis
Source: Front Cardiovasc Med. 2023 Oct 17;10:1149351. doi: 10.3389/fcvm.2023.1149351 (PMC10616249; doi:10.3389/fcvm.2023.1149351)

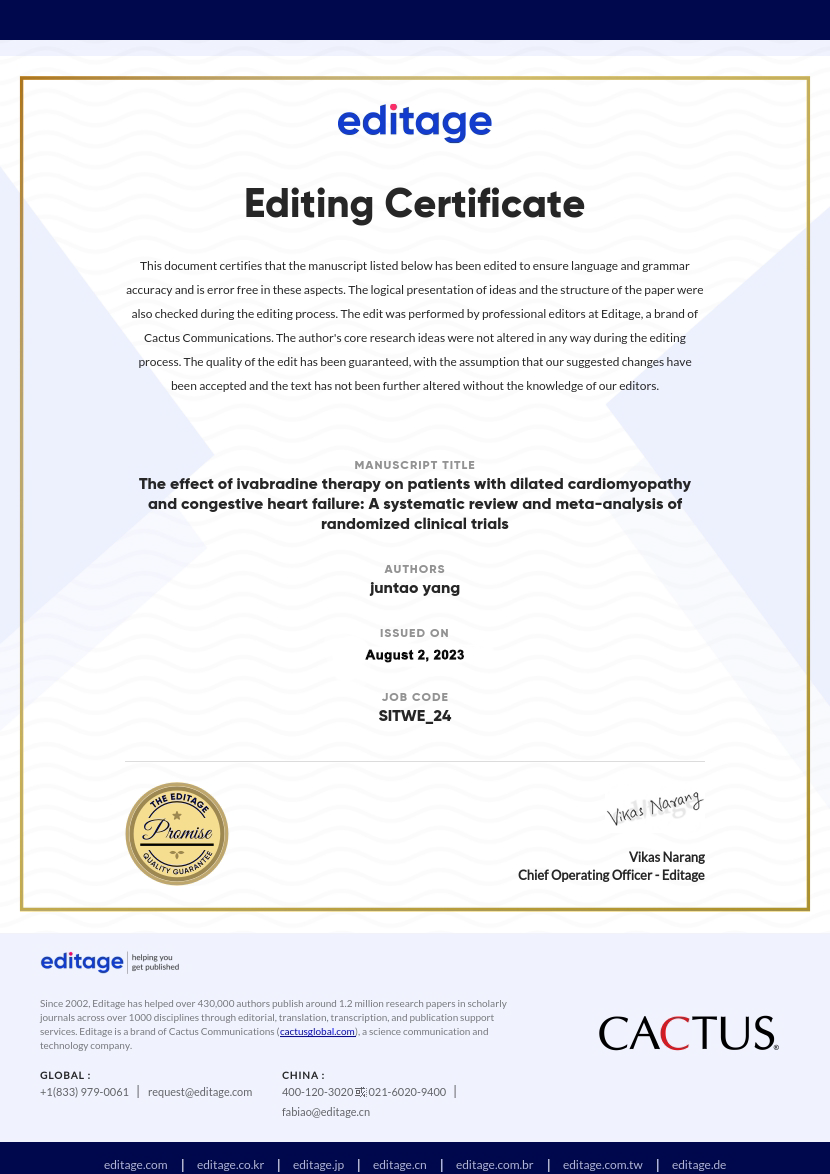

Supplement: Supplementary file 2 [file Image1.tif]
